# Editorial: Special Issue on “Therapeutic Approaches for Cystic Fibrosis”

**DOI:** 10.3390/ijms21186657

**Published:** 2020-09-11

**Authors:** Nicoletta Pedemonte

**Affiliations:** UOC Genetica Medica, IRCCS Giannina Gaslini, 16147 Genova, Italy; nicoletta.pedemonte@unige.it

Cystic fibrosis (CF) is the most common lethal genetic disease in Caucasian populations, occurring in approximately 1 in 3000 newborns worldwide [1]. CF is caused by loss-of-function mutations in the gene encoding for the CF transmembrane conductance regulator (CFTR) protein, acting as a chloride- and bicarbonate-permeable channel in epithelial cells [2]. CF is a multi-organ disease, but the major causes of morbidity and mortality are the respiratory symptoms. In the airways, impaired CFTR activity, and subsequent increased sodium absorption through the epithelial sodium channel, lead to a depletion of the airway surface liquid, increasing mucus viscosity and impairing mucociliary clearance [1]. Bacteria are, therefore, trapped in the viscous mucus layer on top of respiratory epithelial cells, leading to recurrent lung infections and chronic inflammation [1]. The temporal and causal relationships between lung infection and inflammation have not been completely clarified: pivotal studies performed on a CF pig model highlighted a host defense defect in eradicating bacteria within hours of birth, but lack of inflammation [3]. Gray and colleagues [4] demonstrated that neutrophil survival is constitutively increased in CF due to decreased apoptosis, allowing more neutrophils to form neutrophil extracellular trap (NET) that will interact with macrophages and promote inflammation. Finally, macrophages from newborn CF pigs were found to exhibit an increased inflammatory response to a lipopolysaccharide challenge, that may contribute to the onset and progression of CF lung disease [5].

Until 2012, therapies for CF patients were focused on preventing and treating its consequences: airway bacterial colonization, mucociliary clearance deficit, and intestinal malabsorption. However, for most patients, the final life-saving intervention remained lung transplantation. Since 2012, personalized medicine approaches have been introduced into the therapeutic regimens for CF patients carrying specific genotypes [6,7]. In particular, drugs called “potentiators” have been developed to increase the activity of CFTR mutants displaying defective gating of the channel, while compounds acting as “correctors” are able to promote maturation and trafficking to the plasma membrane of misfolded CFTR mutants [6,7]. The increasing availability of the so-called “CFTR modulators”, able to overcome the basic defects due to CFTR mutations, have demonstrated the potential to radically change the progression of the disease. This is the case of the potentiator ivacaftor (Kalydeco^®^; see ref. [8]), or of the recently approved corrector/potentiator combination tezacaftor/elexacaftor/ivacaftor (Trikafta/Kaftrio^®^; see ref. [8]), which appears to be—by far—more effective than the previously developed combinations lumacaftor/ivacaftor (Orkambi^®^; see [8]) and tezacaftor/ivacaftor (Symdeko/Symkevi^®^; see [8]). However, we have to consider that in vitro modulator efficacy does not always reflect their in vivo efficacy due, for example, to the persistent chronic inflammation.

In addition, some CF mutations currently appear less amendable to rescue with CFTR modulators, despite their high efficacy [6,7]. Indeed, an estimated 10% of patients will not be responsive to these drugs, most notably the nonsense mutations which eliminate protein expression, or particular missense mutations [6,7]. In these cases, mutation-independent therapies will be the strategy of choice [6,7].

This Special Issue on “Therapeutic Approaches for Cystic Fibrosis” gathers a collection of 19 original and review articles bringing insights into CFTR biology, CFTR modulators, genotype-agnostic approaches, regulation of airway surface liquid (ASL) homeostasis, antimicrobial and anti-inflammatory agents in the CFTR modulators era, and the role of CFTR dysfunction in cancer.

CFTR proteostatic network plays an important role in CFTR biogenesis, a process that takes place in different cellular compartments and involves interaction with several protein complexes [9]. Estabrooks and Brodsky [10] review the many cellular checkpoints that monitor CFTR biogenesis as it transitions from the endoplasmic reticulum (ER) to the plasma membrane. The authors also discuss the pharmacological modulators of CFTR biogenesis that can repair CFTR, permitting its folding, escape from ERAD, and function at the cell surface, leading to effective treatments for CF, and highlighting future areas of research on the proteostatic control of CFTR [10].

Among the molecular factors regulating CFTR trafficking, stability and degradation that might represent drug targets to improve CFTR rescue, Degrugillier et al. [11] focus on the impact of HspB5 expression and phosphorylation on transport to the plasma membrane, function and stability of F508del-CFTR. The authors show that a phosphomimetic form of HspB5 increases the transport to the plasma membrane, function and stability of F508del-CFTR, with additive effects with CFTR modulators, thus supporting its therapeutic relevance in cystic fibrosis [11].

It has been described that potentiators can destabilize mutant CFTR expressed at the plasma membrane (PM) after rescue with correctors [12,13]. As a strategy to improve F508del-CFTR stability at the PM, Mancini and colleagues [14] used ganglioside GM1, a sphingolipid involved in the functional stabilization of transmembrane proteins. They found that GM1 resides in the same PM microenvironment as CFTR and that, in CF cells, the expression of the mutated channel is accompanied by a decrease in the PM GM1 content [14]. Interestingly, the exogenous administration of GM1 reduced the destabilizing effect of the potentiator VX-770 on rescued CFTR protein expression/function, improving its stabilization [14]. This could represent a starting point for developing innovative therapeutic strategies based on the co-administration of GM1 and CFTR modulators, with the aim of improving F508del-CFTR function.

Among the molecular interactors regulating CFTR membrane retention, particularly important is the Na^+^/H^+^ exchanger regulatory factor isoform-1 (NHERF-1), that binds CFTR to the apical actin cytoskeleton and regulates CFTR membrane retention [15]. Cytoskeleton integrity is critical for the immobilization of CFTR in the plasma membrane. Disease-causing mutations, such as F508del-CFTR, have been linked to the blocking of CFTR association with NHERF-1, resulting in the mistargeting, altered recycling and impaired regulation of the channel [16]. Overexpression of NHERF-1 in F508del-CFTR cells has been shown to stabilize the F508del-CFTR at the plasma membrane and to promote cytoskeleton organization [17]. Thus, CFTR membrane retention, CFTR association with NHERF-1 and cytoskeleton organization are key factors in CF disease mechanisms. As a way to study actin cytoskeleton alteration in CF cells, Carapeto et al. [18] used Atomic Force Microscopy and Force Feedback Microscopy to investigate the mechanical properties of cystic fibrosis bronchial epithelial (CFBE) cells stably transduced with either wild type (wt-) or F508del-CFTR. They found that the expression of mutant CFTR causes a decrease in the cell’s apparent Young (elastic) modulus as compared to the expression of the wt protein [18]. This decrease is likely the consequence of a more disorganized actin cytoskeleton possibly due to a depolymerization of the actin fibers network below the cell membrane, related with the F508del mutation [18].

Understanding the mechanisms exerted by correctors to increase mutant F508del-CFTR expression is essential for the development of new CF therapeutics. To this aim, Amico et al. [19] investigated the activity of four correctors on the mutant F508del and wt CFTR to identify the protein domains whose expression is mostly affected by the action of correctors. Interestingly, three correctors, VX809, VX661 and VX325, seem to specifically improve the expression and the maturation of the mutant CFTR N-half (M1N1, residues 1–633), while the CFTR C-half (M2N2, residues 837–1480) appears to be the region mainly affected by corr4a. These findings provide new insights for the design of new structure-based CF therapeutics [19].

Among the different classes of correctors identified during the last 15 years, iminosugars are endowed of a wide range of biological activities and exhibit excellent drug profile [20]. Iminosugars are sugar analogues that act as inhibitors or enhancers of carbohydrate-processing enzymes [20]. Esposito et al. [21] review current knowledge on this class of compounds, summarizing the evidences that support their potential use as therapeutics for the treatment of CF. Indeed, starting from the example of the marketed drug NBDNJ (N-butyl deoxynojirimycin), a variety of iminosugars have exhibited the capacity to rescue the trafficking of F508del-CFTR, either alone or in combination with other correctors [22]. In addition, iminosugars have shown also activity as anti-inflammatory agents in CF lung disease [23].

The treatment of F508del homozygous CF patients with the lumacaftor/ivacaftor combination result in some amelioration of the respiratory function [8]. However, a great variability in the clinical response is also observed. Favia et al. [24] evaluated the response to Orkambi^®^ in a small cohort of F508del/F508del patients in terms of clinical and laboratory parameters, including ex vivo CFTR activity in mononuclear cells (MNCs), during a 12-month treatment. Patients responded with an increase in percent predicted forced expiratory volume in 1 s (FEV1%) and body mass index (BMI) [24]. Sweat chloride and CFTR-dependent chloride efflux were found to decrease and increase, respectively, as compared with pre-therapy values [24]. Interestingly, CFTR and BMI showed a statistically significant correlation during Orkambi^®^ treatment, while clustering analysis showed that CFTR, BMI, sweat chloride, FEV1%, and white blood cell total counts were strongly associated [24]. Thus, the authors suggest to consider the use of CFTR-dependent chloride efflux in MNCs as a sensitive outcome measure of Orkambi^®^ treatment in CF patients [24].

Although the results obtained so far by the already approved CFTR modulators, there is still an urgent need for novel drugs endowed with higher potency and efficacy in terms of mutant CFTR rescue. How is it possible to design such optimized modulator drugs? Rusnati and colleagues [25] answer to this question by proposing computational methods and biosensors, two methodologies that have become indispensable tools in the process of drug discovery for many important human pathologies. In their review article, the authors give an overview of the available structures and computational models of CFTR and of the biosensors, biochemical and cell-based assays already used in CF-oriented studies, providing some insights about how to integrate these tools as to improve the efficiency of the drug discovery process targeted to CFTR [25].

Among the mutation-agnostic approaches proposed to overcome the basic defect in CF, a major field of research is based on the exploitation of alternative targets [7]. Of particular interest is the calcium-dependent chloride channel TMEM16A, which is upregulated during inflammatory lung disease [26]. TMEM16A activation would improve hydration of the airway mucus and increase mucociliary clearance [26]. However, TMEM16A is also essential for mucus production and/or secretion, and appears to promote mucus secretion during inflammatory airway disease [27]. Danahay and Gosling [28] summarize the current progress with CFTR-independent approaches to restoring mucosal hydration, with particular emphasis on modulation of TMEM16A and the controversy regarding whether it should be positively or negatively modulated. This is discussed also in light of the recent report describing for the first time TMEM16A potentiators and their positive effects upon epithelial fluid secretion and mucus clearance [28].

Pharmacological activators and inhibitors of TMEM16A developed so far have been identified using different cell models. For this reason, Centeio et al. [29] investigated and compared the mechanisms of activation and inhibition of endogenous and overexpressed TMEM16A and analyzed potential off-target effects in different cell types. The results of their study suggest that overexpressed TMEM16A might have a better accessibility to intracellular Ca^2+^, causing spontaneous activity at basal intracellular Ca^2+^ concentrations [29]. As a consequence, small molecules may potentiate pre-stimulated TMEM16A currents, but otherwise fail to activate silent endogenous TMEM16A [29].

Another strategy to replace the defective transport activity elicited by CFTR in CF airway epithelia is based on the exploitation of small molecules capable of facilitating the transmembrane transport of chloride and bicarbonate, the so-called “anionophores” [30]. A study by Gianotti and colleagues [31] provide evidences that treatment of primary bronchial epithelia with non-toxic doses of synthetic anionophores improved the periciliary fluid composition, reducing the fluid re-absorption, correcting the ASL pH and reducing the viscosity of the mucus. This work supports the role of anionophores as promising drug candidates for CF therapy.

Gene therapy still holds the promise of a permanent solution to genetic diseases including CF. In the last years, the development of refined technologies allowing site specific modification with programmable nucleases has facilitated genome manipulation, offering diversified strategies to reverse mutations. Maule et al. [32] give a comprehensive overview of the advancement of gene therapy, from therapeutic nucleic acids to genome editing techniques, and their applications for the development of experimental models valuable for the advancement of CF therapies.

Mucociliary clearance is a component of the innate immunity and plays a pivotal role in protecting the host from inhaled pathogens [33]. Mucociliary clearance is mediated by the movements of cilia bathing in the ASL on the surface of airway epithelium [33]. ASL volume and composition is maintained by the coordinated function of ion channels, transporters, and pumps that regulate transcellular and paracellular movement of ions and water [33,34]. ASL volume and composition are important for ciliary beating and mucus hydration [33,34]. A review by Mitash et al. [35] discuss the role of microRNA (miRNA), a class of non-coding, short, single-stranded RNA regulating gene expression by post-transcriptional mechanisms, in ASL homeostasis and host–pathogen interactions in the airway. Indeed, miRNAs have been recognized as essential regulators of ion channels and transporters involved in ASL homeostasis [35]. The authors also discuss concepts for miRNA-directed therapy [35].

Inhaled sodium bicarbonate has been proposed as an adjuvant therapy in CF due to its mucolytic and bacteriostatic properties [36]. A study by Gróf et al. [37] investigated its direct effect on CFBE cells expressing wt- or F508del-CFTR, co-cultured with human vascular endothelial cells. Their results show that sodium bicarbonate significantly decreased the more-alkaline intracellular pH of the mutant CFBE cells, suggesting a direct therapeutic effect on the bronchial epithelium [37].

Impaired mucociliary clearance and subsequent recurrent and chronic bacterial infections are associated to an excess of neutrophilic inflammation [38]. While neutrophil serine proteases are a crucial part of the innate host defence to infection, a surplus of neutrophil elastase (NE) is understood to create a net destructive effect [39]. A review article by Hunt and collaborators [40] discuss the role of the antiprotease alpha-1 antitrypsin (A1AT) in the control of NE protease activity. A1AT is ineffective in the CF lung, due to the huge imbalance of NE levels [40]. The authors summarize the therapeutic strategies to boost levels of protective antiproteases such as A1AT in the lung as an attractive research strategy to limit the damage from excess protease activity [40]. Particularly interesting are the miRNA targeted therapies exploiting inhibition of miRNAs targeting the *SERPINA1* (A1AT-encoding gene) mRNA, as a strategy to drive endogenous A1AT production and thus reinforcing the antiprotease shield of the CF lung [40].

Treatment burden sustained by CF patients, side effects of current medications, and recent advances in CFTR modulator drugs have highlighted the need to develop novel therapies targeting the inflammatory component that drives CF lung damage. In addition, current issues with standard treatments emphasize the need for directed lung therapies that could minimize systemic side effects. These topics are extensively discussed in a review article by Giacalone et al. [41], providing an overview of the current treatments used to target immune cells in the lungs, and giving valuable insights into the potential benefits and caveats of novel therapeutic strategies.

Nontuberculous mycobacteria (NTM) have recently emerged as important pathogens among CF patients worldwide [42]. Among NTM, Mycobacterium abscessus is particularly worrisome [43]. A review by Degiacomi and colleagues [44] summarize current knowledge on this pathogen, including its physiology, transmission, current drug therapy as well as possible alternative strategies, and discuss recent evidences linking CFTR dysfunction to immune control of M. abscessus infections.

The improvements in drug therapies for CF has led to an increase in life expectancy for CF individuals, that has led to a number of medical consequences for the CF population. Particular comorbidities that are age related include the so-called CF-related disease (CFRD), bone disease and arthropathy, which may benefit from treatment with CFTR modulators [45]. Surprisingly, obesity might also be a potential issue considering that CFTR modulators lead to increased body weight; thus, standard diets will need to be modified [45]. Importantly, ageing of CF patients has also highlighted an unexpectedly elevated predisposition to cancer, firmly established by recent studies involving large cohorts of patients [46]. In this Special Issue, two review articles discuss this novel challenge emerging at the forefront of CF disease. The first review, by Amaral et al. [47], provides state-of-the-art descriptions on the key roles of CFTR in fundamental cellular processes such as foetal development, epithelial differentiation/polarization, and regeneration, as well as in epithelial–mesenchymal transition, highlighting how they can contribute to novel therapeutic strategies. The second review, by Scott and colleagues [48], summarizes epidemiology of cancer in CF individuals and discusses the possible mechanisms underlying the actions of CFTR as a tumor suppressor: dysregulation of Wnt/β-catenin signaling, disruption of intestinal stem cell homeostasis and intestinal barrier integrity, intestinal dysbiosis, immune cell infiltration, stress responses, and intestinal inflammation. Interestingly, the authors also comment on the intriguing hypothesis that CFTR modulators could be repurposed for gastrointestinal cancers [48].

In conclusion, this Special Issue on “Therapeutic Approaches for Cystic Fibrosis” provides an overview of the new therapeutic approaches that are being attempted in the CF field. These include the development of: (1) novel, more effective CFTR modulators, especially for those mutants not responsive to current modulators; (2) pharmacological modulators of new therapeutic targets to promote the rescue of mutant CFTR and its expression and stabilization on the plasma membrane; (3) new strategies to restore chloride/bicarbonate secretion and the correct homeostasis of the ASL; (4) new specific immunomodulatory drugs optimized for CF lung; (5) new antimicrobial drugs for emerging pathogens. While, on one hand, the current therapies have allowed an increased life span for people with CF, on the other hand this has led to the emergence of new medical challenges related to CFTR dysfunction. Of particular relevance is the role of CFTR, and the consequences of its impairment, in carcinogenesis. These findings pave the way for new lines of research, which might positively impact many pathologies, even beyond CF.

## References

[1] Ratjen F., Döring G. (2003). Cystic fibrosis. Lancet.

[2] Hwang T.C., Yeh J.T., Zhang J., Yu Y.C., Yeh H.I., Destefano S. (2018). Structural mechanisms of CFTR function and dysfunction. J. Gen. Physiol..

[3] Stoltz D.A., Meyerholz D.K., Pezzulo A.A., Ramachandran S., Rogan M.P., Davis G.J., Hanfland R.A., Wohlford-Lenane C., Dohrn C.L., Bartlett J.A. (2010). Cystic fibrosis pigs develop lung disease and exhibit defective bacterial eradication at birth. Sci. Transl. Med..

[4] Gray R.D., Hardisty G., Regan K.H., Smith M., Robb C.T., Duffin R., Mackellar A., Felton J.M., Paemka L., McCullagh B.N. (2018). Delayed neutrophil apoptosis enhances NET formation in cystic fibrosis. Thorax.

[5] Paemka L., McCullagh B.N., Abou Alaiwa M.H., Stoltz D.A., Dong Q., Randak C.O., Gray R.D., McCray P.B. (2017). Monocyte derived macrophages from CF pigs exhibit increased inflammatory responses at birth. J. Cyst. Fibros..

[6] Amaral M.D. (2015). Novel personalized therapies for cystic fibrosis: Treating the basic defect in all patients. J. Intern. Med..

[7] Li H., Pesce E., Sheppard D.N., Singh A.K., Pedemonte N. (2018). Therapeutic approaches to CFTR dysfunction: From discovery to drug development. J. Cyst. Fibros..

[8] Gramegna A., Contarini M., Aliberti S., Casciaro R., Blasi F., Castellani C. (2020). From Ivacaftor to Triple Combination: A Systematic Review of Efficacy and Safety of CFTR Modulators in People with Cystic Fibrosis. Int. J. Mol. Sci..

[9] Sondo E., Pesce E., Tomati V., Marini M., Pedemonte N. (2017). RNF5, DAB2 and Friends: Novel Drug Targets for Cystic Fibrosis. Curr. Pharm. Des..

[10] Estabrooks S., Brodsky J.L. (2020). Regulation of CFTR Biogenesis by the Proteostatic Network and Pharmacological Modulators. Int. J. Mol. Sci..

[11] Degrugillier F., Aissat A., Prulière-Escabasse V., Bizard L., Simonneau B., Decrouy X., Jiang C., Rotin D., Fanen P., Simon S. (2020). Phosphorylation of the Chaperone-Like HspB5 Rescues Trafficking and Function of F508del-CFTR. Int. J. Mol. Sci..

[12] Veit G., Avramescu R.G., Perdomo D., Phuan P.W., Bagdany M., Apaja P.M., Borot F., Szollosi D., Wu Y.S., Finkbeiner W.E. (2014). Some gating potentiators, including VX-770, diminish F508del-CFTR functional expression. Sci. Transl. Med..

[13] Cholon D.M., Quinney N.L., Fulcher M.L., Esther C.R., Das J., Dokholyan N.V., Randell S.H., Boucher R.C., Gentzsch M. (2014). Potentiator ivacaftor abrogates pharmacological correction of F508del CFTR in cystic fibrosis. Sci. Transl. Med..

[14] Mancini G., Loberto N., Olioso D., Dechecchi M.C., Cabrini G., Mauri L., Bassi R., Schiumarini D., Chiricozzi E., Lippi G. (2020). GM1 as Adjuvant of Innovative Therapies for Cystic Fibrosis Disease. Int. J. Mol. Sci..

[15] Farinha C.M. (2018). CFTR and Cystic Fibrosis. CFTR and Cystic Fibrosis.

[16] Voltz J.W., Weinman E.J., Shenolikar S. (2001). Expanding the role of NHERF, a PDZ-domain containing protein adapter, to growth regulation. Oncogene.

[17] Favia M., Guerra L., Fanelli T., Cardone R.A., Monterisi S., Di Sole F., Castellani S., Chen M., Seidler U., Reshkin S.J. (2010). Na_+_/H_+_ exchanger regulatory factor 1 overexpression-dependent increase of cytoskeleton organization is fundamental in the rescue of F508del cystic fibrosis transmembrane conductance regulator in human airway CFBE41o-cells. Mol. Biol. Cell.

[18] Carapeto A.P., Vitorino M.V., Santos J.D., Ramalho S.S., Robalo T., Rodrigues M.S., Farinha C.M. (2020). Mechanical Properties of Human Bronchial Epithelial Cells Expressing Wt- and Mutant CFTR. Int. J. Mol. Sci..

[19] Amico G., Brandas C., Moran O., Baroni D. (2019). Unravelling the Regions of Mutant F508del-CFTR More Susceptible to the Action of Four Cystic Fibrosis Correctors. Int. J. Mol. Sci..

[20] Compain P., Martin O.R. (2007). Iminosugars: From Synthesis to Therapeutic Applications.

[21] Esposito A., D’Alonzo D., De Fenza M., De Gregorio E., Tamanini A., Lippi G., Dechecchi M.C., Guaragna A. (2020). Synthesis and Therapeutic Applications of Iminosugars in Cystic Fibrosis. Int. J. Mol. Sci..

[22] Norez C., Noel S., Wilke M., Bijvelds M., Jorna H., Melin P., Dejonge H., Becq F. (2006). Rescue of functional delF508-CFTR channels in cystic fibrosis epithelial cells by the α-glucosidase inhibitor miglustat. FEBS Lett..

[23] Dechecchi M.C., Nicolis E., Norez C., Bezzerri V., Borgatti M., Mancini I., Rizzotti P., Ribeiro C.M.P., Gambari R., Becq F. (2008). Anti-inflammatory effect of miglustat in bronchial epithelial cells. J. Cyst. Fibros.

[24] Favia M., Gallo C., Guerra L., De Venuto D., Diana A., Polizzi A.M., Montemurro P., Mariggiò M.A., Leonetti G., Manca A. (2020). Treatment of Cystic Fibrosis Patients Homozygous for *F508del* with Lumacaftor-Ivacaftor (Orkambi^®^) Restores Defective CFTR Channel Function in Circulating Mononuclear Cells. Int. J. Mol. Sci..

[25] Rusnati M., D’Ursi P., Pedemonte N., Urbinati C., Ford R.C., Cichero E., Uggeri M., Orro A., Fossa P. (2020). Recent Strategic Advances in CFTR Drug Discovery: An Overview. Int. J. Mol. Sci..

[26] Pedemonte N., Galietta L.J. (2014). Structure and function of TMEM16 proteins (anoctamins). Physiol. Rev..

[27] Kunzelmann K., Ousingsawat J., Cabrita I., Doušová T., Bähr A., Janda M., Schreiber R., Benedetto R. (2019). TMEM16A in Cystic Fibrosis: Activating or Inhibiting?. Front. Pharm..

[28] Danahay H., Gosling M. (2020). TMEM16A: An Alternative Approach to Restoring Airway Anion Secretion in Cystic Fibrosis?. Int. J. Mol. Sci..

[29] Centeio R., Cabrita I., Benedetto R., Talbi K., Ousingsawat J., Schreiber R., Sullivan J.K., Kunzelmann K. (2020). Pharmacological Inhibition and Activation of the Ca^2+^ Activated Cl^−^ Channel TMEM16A. Int. J. Mol. Sci..

[30] Li H., Salomon J.L., Sheppard D.N., Mall M.A., Galietta L.J.V. (2017). Bypassing CFTR dysfunction in cystic fibrosis with alternative pathways for anion transport. Curr. Opin. Pharm..

[31] Gianotti A., Capurro V., Delpiano L., Mielczarek M., García-Valverde M., Carreira-Barral I., Ludovico A., Fiore M., Baroni D., Moran O. (2020). Small Molecule Anion Carriers Correct Abnormal Airway Surface Liquid Properties in Cystic Fibrosis Airway Epithelia. Int. J. Mol. Sci..

[32] Maule G., Arosio D., Cereseto A. (2020). Gene Therapy for Cystic Fibrosis: Progress and Challenges of Genome Editing. Int. J. Mol. Sci..

[33] Widdicombe J.H. (2002). Regulation of the depth and composition of airway surface liquid. J. Anat..

[34] Tarran R., Button B., Boucher R.C. (2006). Regulation of normal and cystic fibrosis airway surface liquid volume by phasic shear stress. Annu. Rev. Physiol..

[35] Mitash N., Donovan J.E., Swiatecka-Urban A. (2020). The Role of MicroRNA in the Airway Surface Liquid Homeostasis. Int. J. Mol. Sci..

[36] Pezzulo A.A., Tang X.X., Hoegger M.J., Abou Alaiwa M.H., Ramachandran S., Moninger T.O., Karp P.H., Wohlford-Lenane C.L., Haagsman H.P.v.E.M., Bánfi B. (2012). Reduced airway surface pH impairs bacterial killing in the porcine cystic fibrosis lung. Nature.

[37] Gróf I., Bocsik A., Harazin A., Santa-Maria A.R., Vizsnyiczai G., Barna L., Kiss L., Fűr G., Rakonczay Z., Ambrus R. (2020). The Effect of Sodium Bicarbonate, a Beneficial Adjuvant Molecule in Cystic Fibrosis, on Bronchial Epithelial Cells Expressing a Wild-Type or Mutant CFTR Channel. Int. J. Mol. Sci..

[38] Gifford A.M., Chalmers J.D. (2014). The role of neutrophils in cystic fibrosis. Curr. Opin. Hematol..

[39] Taggart C., Mall M.A., Lalmanach G., Cataldo D., Ludwig A., Janciauskiene S., Heath N., Meiners S., Overall C.M., Schultz C. (2017). Protean proteases: At the cutting edge of lung diseases. Eur. Respir. J..

[40] Hunt A.M., Glasgow A.M., Humphreys H., Greene C.M. (2020). Alpha-1 Antitrypsin—A Target for MicroRNA-Based Therapeutic Development for Cystic Fibrosis. Int. J. Mol. Sci..

[41] Giacalone V.D., Dobosh B.S., Gaggar A., Tirouvanziam R., Margaroli C. (2020). Immunomodulation in Cystic Fibrosis: Why and How?. Int. J. Mol. Sci..

[42] Skolnik K., Kirkpatrick G., Quon B.S. (2016). Nontuberculous Mycobacteria in Cystic Fibrosis. Curr. Treat. Options Infect. Dis..

[43] Martiniano S.L., Nick J.A., Daley C.L. (2019). Nontuberculous Mycobacterial Infections in Cystic Fibrosis. Thorac. Surg. Clin..

[44] Degiacomi G., Sammartino J.C., Chiarelli L.R., Riabova O., Makarov V., Pasca M.R. (2019). *Mycobacterium abscessus*, an Emerging and Worrisome Pathogen among Cystic Fibrosis Patients. Int. J. Mol. Sci..

[45] Ronan N.J., Elborn J.S., Plant B.J. (2017). Current and emerging comorbidities in cystic fibrosis. Presse Med..

[46] Miller A.C., Comellas A.P., Hornick D.B., Stoltz D.A., Cavanaugh J.E., Gerke A.K., Welsh M.J., Zabner J., Polgreen P.M. (2020). Cystic fibrosis carriers are at increased risk for a wide range of cystic fibrosis-related conditions. Proc. Natl. Acad. Sci. USA.

[47] Amaral M.D., Quaresma M.C., Pankonien I. (2020). What Role Does CFTR Play in Development, Differentiation, Regeneration and Cancer?. Int. J. Mol. Sci..

[48] Scott P., Anderson K., Singhania M., Cormier R. (2020). Cystic Fibrosis, CFTR, and Colorectal Cancer. Int. J. Mol. Sci..

